# Exploring Culinary Methods to Reduce Sodium Intake: The Impact of Flavorings and Salt Addition Timing in Boiled Chicken

**DOI:** 10.1155/ijfo/3703692

**Published:** 2025-06-06

**Authors:** Raphael Monod, Thierry Thomas-Danguin, Henriette L. de Kock

**Affiliations:** ^1^Centre for Taste, Smell, and Feeding Behavior (CSGA), INRAE, CNRS, Institut Agro Dijon, Universite Bourgogne Europe, Dijon, France; ^2^Universite Clermont Auvergne, INRAE, QuaPA Research Unit, Saint-Genes-Champanelle, France; ^3^Department of Consumer and Food Sciences, University of Pretoria, Hatfield, South Africa

**Keywords:** domestic practices, OITE, salt reduction, saltiness intensity, table salt

## Abstract

The overconsumption of salt is a social concern and has consequences for human health. Discretionary salt contributes to salt intake but has received very little attention thus far, and recommendations do not precisely targeted discretionary salt. This study investigated how different culinary practices affect the saltiness of chickens. Chicken breasts were boiled in a standard homestyle bouillon (broth). Table salt was added to the broth or on the plate after cooking. Two salt concentrations, regular (6.5 mmol of Na^+^ per 100 g of cooked chicken) and low (4.1 mmol of Na^+^ per 100 g of cooked chicken), were compared. Additionally, we applied the following flavor treatments: rosemary, smoked bacon, and smoked garlic. The saltiness, sweetness, sourness, bitterness, and overall aroma of the warm chicken samples were evaluated by 158 untrained consumers. Saltiness adequacy was measured on a just-about-right (JAR) scale. Based on the results, no significant differences in saltiness intensity were observed between the two salting practices (*p* > 0.2). Regarding the flavor additions, a significant odor-induced increase in saltiness was observed when smoked bacon flavoring was combined with salting on the plate (*p* = 0.02). The JAR results indicated that adding smoked garlic flavoring to the broth allows a 33% reduction in salt content without compromising taste, suggesting that smoked garlic has a strong odor-induced saltiness enhancement effect and may be a viable option for salt reduction strategies. This study provides a basis for improving discretionary salt use practices that can be easily adopted by consumers, potentially aiding in reducing salt intake from chicken dishes without compromising flavor, and offering insights that may apply to other meat types.

## 1. Introduction

Research has shown that sodium intake, primarily from salt (NaCl) consumption, is a major factor positively associated with high blood pressure in a dose-response relationship—the lower the salt intake, the greater the reduction in blood pressure [1]. While sodium intake plays a significant role, other contributors to the prevalence of hypertension and cardiovascular diseases (CVDs) include potassium intake, overall diet, and genetic predisposition [2]. Hypertension is a major risk factor for CVDs. Fifteen million people between 30 and 69 years of age die annually from noncommunicable diseases, mostly from CVDs. More than three-quarters of these deaths occur in low- and middle-income countries [3]. Therefore, the World Health Organization (WHO) recommends lowering salt intake to 5 g/day [4]. In South Africa, the mean per capita salt intake was estimated to be between 6 and 11 g per day in 2015 [5]. Although this estimation is based on limited data and reflects variability due to different sources or methodologies used in estimating salt intake, it is clear that South Africans consume excess salt [5]. The overconsumption of salt poses significant risks to the already burdened South African health system [6]. To address this issue, public health campaigns aimed at raising awareness about salt intake, the mandatory reformulation of recipes, and the promotion of alternative flavorings are important strategies. The South African government implemented a mandatory national regulation to reduce salt consumption in 2013. This regulation targeted 13 food categories, including bread, stock cubes, and processed meats. It established phased salt reduction benchmarks for 2016 (Phase 1) and 2019 (Phase 2). By 2018/early 2019, it was estimated that the regulation had resulted in a reduction of 1.2 g of salt per day compared to the 2013–2016 period [7]. Further reductions are expected following the 2019 mandatory salt limits in commercial foods. Salt is consumed from processed foods, discretionary sources, and natural food sources [8]. Charlton et al. [9] suggested that salt added to food at the table and in cooking comprises up to 40% of the total salt intake in South Africa, which is equivalent to a daily added salt amount of 4.3 g. Moreover, the use of table salt may increase when manufactured products lack sufficient saltiness. For example, when eating chicken stews cooked with low salt stock cubes, some people add table salt and even fully replace the salt removed [10]. Hence, discretionary salt may remain an important contributor to dietary salt intake [11].

Several strategies have been used to enhance the saltiness of food. For example, Emorine et al. [12] combined the heterogeneous spatial distribution of flavors and aroma-taste interactions to increase the saltiness of hot snacks. Ham, which has a salt-associated aroma, enhanced saltiness regardless of the spatial distribution of salt and aroma. Moreover, products with heterogeneous salt distributions were perceived as saltier. Furthermore, pulsatile stimulation can enhance salt perception [13], possibly by reducing adaptation [14]. The heterogeneous distribution of salt (NaCl) is a promising way to generate the pulsatile stimulation by creating unsalted (or low) salted areas and highly salted areas in the same food product. The uneven distribution of salt leads to a significantly greater perception of salt intensity for hot snacks [15], pizza [16], bread [17], and meat [18].

Broths are used to add flavor to a dish. Broth consumption in sub-Saharan countries is high, contributing to salt intake above recommended levels [19]. When broth is reformulated with the objective of exploiting cross-modal interactions (such as aroma-taste interactions), the overall flavor intensity and consumer satisfaction can be maintained while reducing salt levels [20]. Effectively, certain odors are linked to taste and can provoke a phenomenon known as odor-induced taste enhancement (OITE) [21, 22]. In the case of saltiness, OITE has been described in salty water using soy sauce aroma [21, 23], smoked bacon or smoked garlic [24], bacon, sardine or anchovy [25], and sardine [26]. OITE has also been achieved in complex liquids through the application of beef stock in a green pea soup [27] and savory aroma compounds, such as beef flavor, in beef broths [28]. Most studies focusing on OITE have used water with salt and aromas [27]. In two studies, OITE was tested in a cheese model using sardine aroma [29, 30]. Overall, a richer flavor system can compensate for a reduced salt content [31]. Flavor can be added by using salty congruent aromas as described previously or by using herbs and spices [32]. It has been reported that saline solutions flavored with Mediterranean herbs and spices (i.e., *Helichrysum*, rosemary, licorice, fennel seeds, and myrtle leaves) may enhance saltiness perception compared to unflavored salt solutions [33]. For instance, patients with hyposmia perceived saline solutions made with these flavored salts, which contained approximately 15% less salt, as similarly intense in saltiness and equally pleasant to pure salt solutions [34]. Herbs and spices have been used in a wide variety of food categories, including meat-based meals. For example, garlic, rosemary, oregano or sage, and a blend of garlic, rosemary, and sage can be added as natural flavor enhancers in reduced-salt chicken supremes (a boneless, skin-on breast of chicken) [35]. Consumers added less salt to chicken noodle soup when the perceived herb flavor increased [36]. The addition of a spice blend (Southwest chipotle, basil, pepper, and garlic) to a chicken pasta meal reduced the salt content by 50% [37]. Recently, Petersen et al. [38] used herbs and spices to enhance the flavor of commonly consumed foods. In that study, consumers significantly preferred a chicken in a cream sauce consisting of onion, garlic powders, ground mustard seed, black pepper, parsley flakes, and dill (in terms of overall liking, appearance, and flavor) over the original recipe (which was a chicken in a cream sauce made of whole milk, salt, salted butter, and flour with no flavorings).

In this study, we aimed to assess the impact of different flavoring and salting practices on the enhancement of salt perception in boiled chicken, a commonly prepared home-cooked dish in South Africa. Chicken breasts were cooked in standard homestyle broth. The following methods of adding discretionary salt were investigated: during boiling in the broth and on the plate after cooking. We hypothesized that salting the chicken on the plate would allow salt crystals to remain on the surface of the meat, leading to a more heterogeneous salt distribution and improved salt perception. Additionally, three flavor treatments were applied to the broth, that is, the addition of rosemary, smoked bacon, or smoked garlic flavorings. We postulated that these salt-free flavorings would produce odor-induced saltiness enhancement.

## 2. Materials and Methods

### 2.1. Preparation of Chicken Samples

Fresh boneless and skinless chicken breasts were purchased from a local supermarket (Uitkyk Vleismark, South Africa) and stored at −20°C. They were thawed in a cold room maintained at 3°C–5°C for 24 h prior to cooking and analysis. The partially thawed chicken breasts were cut into 1.5 cm^3^ cubes (each cube's average weight = 5.1 g, standard deviation = 0.5 g). Forty chicken cubes (total weight = 204.9 g, standard deviation = 7.9 g) were cooked in vegetable broth for 6 min before analysis. The vegetable broth consisted of natural spring water (1 L, Aquelle, South Africa) and dehydrated onion, leek, celery, and carrot powders (5 g each, HerboDirect, France).

### 2.2. Sodium Content Analyses

**Figure 1:**
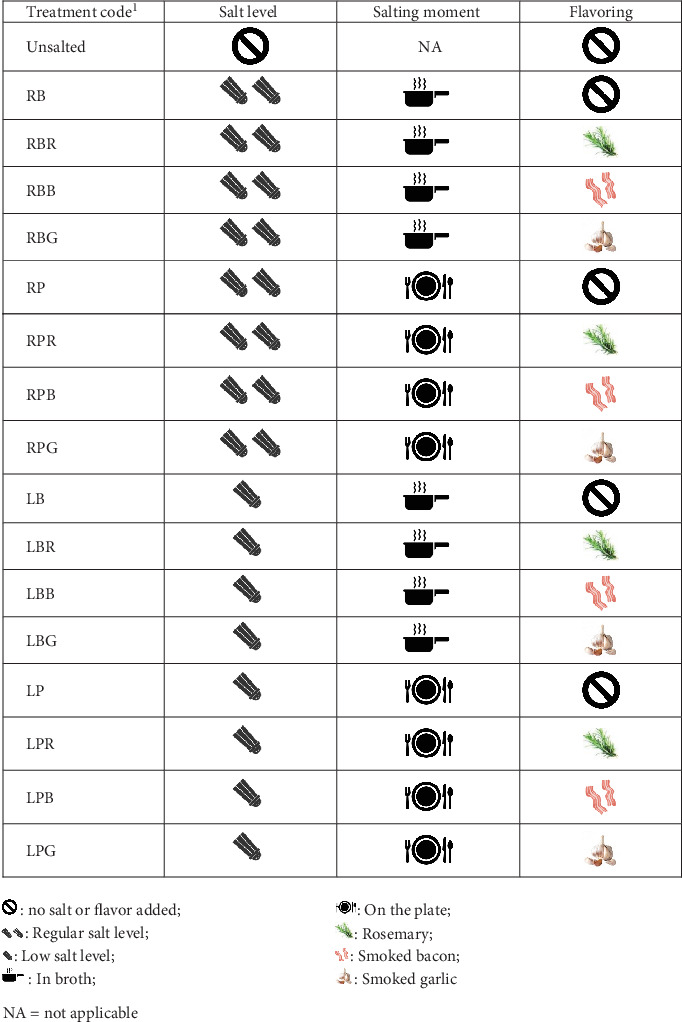
Treatment characteristics.

Inductively coupled plasma–mass spectrometry (ICP–MS) (Agilent, Application Notes 5990-4539EN) was used to determine the total sodium content for the *Unsalted*, *RB*, and *LB* treatments (Figure 1). ICP–MS measurements were conducted by an external company (SGS, Cape Town, South Africa).

In addition, ionic chromatography (IC) analyses were performed in France (CSGA, Dijon) to determine the maximum in-water releasable sodium content in several samples. The IC analyses were performed using skinless and boneless chicken breasts purchased from a local supermarket (Carrefour, Dijon, France) and Evian mineral water (Danone, France) as the cooking water. The same protocol was used to prepare and boil the chicken samples in South Africa and France. *Unsalted*, *RB*, *RP*, *LB*, and *LP* treatments (Figure 1) were analyzed in triplicate using IC. Unsalted chickens with smoked garlic, smoked bacon, or rosemary were also analyzed in triplicate by IC to measure the influence of the flavor on the in-water releasable sodium content of the boiled chicken.

To perform IC analyses and simulate in-mouth maximal deconstruction of the food matrix, chicken pieces were homogenized after the preparation process using an Ultra-Turrax (Ika T25D, Germany) for 1 min at 15,000 rpm. A known volume of Milli-Q water (Millipore SAS, Molsheim, France) was added to facilitate the mixing. Afterward, the chicken puree was centrifuged (Beckman Coulter, 20°C, 30 min, acceleration maximum, deceleration minimum) for 30 min at 15,000 × g to separate the water phase containing the releasable sodium. Finally, the supernatant was collected, filtered (pore size 0.45 *μ*m, CIL, Sainte-Foy-la-Grande, France), diluted, and poured into an HPLC vial.

An ion chromatograph ICS-3000 (Dionex, Sunnyvale, CA, USA) was used to determine the sodium concentration in the chicken samples. The ionic chromatograph was equipped with a conductivity detector, a guard column Dionex IonPac CG12A (5 *μ*m particle size, 3 × 30 mm), a separating column CS12A (5 *μ*m particle size, 3 × 150 mm), and a cation self-regenerating suppressor (CSRS ULTRA II 2 mm) via electrochemical methods. The eluent was 11 mmol L^−1^ of sulfuric acid (H_2_SO_4_) at a flow rate of 0.5 mL·min^−1^. The pressure in the system was approximately 1500 psi (104 bars). The eluent was degassed with nitrogen (Gaz N_2_) at a pressure of 2.5 bars in a bottle. The filtered sample was placed in a vial (Batch #420761 PN 50026635 V2-2, USA), thermocontrolled at 10°C by an autosampler (AS), and injected into a 10-*μ*L sample loop of the Dionex ICS-3000 system. A calibration curve was made before every analysis with solutions at increasing NaCl concentrations. The sodium standard solution was prepared by dissolving 125.3 mg of NaCl (CAS 7647-14-5 Sigma-Aldrich S9625-1 kg #SLBW8510) in 200 mL of ultrapure water (18.2 M*Ω*·cm) to yield an assay concentration of 10.7 mM (= 626.5 mg/L). The stock solution was diluted from 0.06 to 2.1 mM (3–125 mg/L) in ultrapure water. The data were acquired by using Chromeleon chromatographic data station software (Version 7.2) installed on a Dell computer.

### 2.3. Culinary Process

Iodized fine sea salt (La Baleine, France) was added either at 12 g/L in broth (regular level, treatments *RB*, *RBR*, *RBB*, *RBG*, *RP*, *RPR*, *RPB*, and *RPG*; Figure 1) equivalent to 205 mmol/L of Na^+^ or at 6 g/L in broth (low level, 50% sodium reduction, treatments *LB*, *LBR*, *LBB*, *LBG*, *LP*, *LPR*, *LPB*, and *LPG*; Figure 1) equivalent to 103 mmol/L of Na^+^. No salt was added to the *Unsalted* treatment. The regular level was chosen based on domestic recipes and after preliminary tests to elicit a just-about-right (JAR) saltiness perception.

Rosemary fluid extract (3 mL/L, Aroma-Zone, France), smoked bacon (2 g/L, bacon smoked flexarome, 880,501 FB542, Firmenich, Switzerland), or smoked garlic (8 g/L, Ducros, France) were used as a flavor. Flavorings were added in these amounts to elicit an equal flavor intensity during consumption (this was tested internally). The smoked bacon and the smoked garlic were added at the beginning of cooking (treatments *RBB*, *RPB*, *LBB*, and *LPB* and *RBG*, *RPG*, *LBG*, and *LPG*, respectively; Figure 1). The rosemary fluid extract was added after cooking; as the fluid extract is sensitive to heat, the extract could not be added to the water at the beginning of the cooking process (treatments *RBR*, *RPR*, *LBR*, and *LPR*; Figure 1). Treatments without flavoring were tested at the following levels: no salt (*Unsalted*), regular (*RB* and *RP*; Figure 1), and low (*LB* and *LP*; Figure 1).

Salt was added either at the beginning of the cooking process, later referred to as “In broth” (treatments *RB*, *RBR*, *RBB*, and *RBG* and *LB*, *LBR*, *LBB*, and *LBG*; Figure 1), or after cooking, referred to as “On the plate” (treatments *RP*, *RPR*, *RPB*, and *RPG* and *LP*, *LPR*, *LPB*, and *LPG*; Figure 1), at the two levels of salting. After 6 min of boiling, the chicken cubes (*m* = 147.5 g, SD = 12.9 g, total weight of the 40 cooked pieces after draining) were drained and stored in a bain-marie (the water temperature of the bain-marie was 79°C) before being served. The *RP*, *RPR*, *RPB*, and *RPG* and *LP*, *LPR*, *LPB*, and *LPG* treatments (Figure 1) were salted in the bain-marie.

### 2.4. Sensory Analysis

Sensory analysis was performed with two different groups of untrained consumers (Group 1 and Group 2; Table 1). Participants were recruited using an internal database and were unfamiliar with the specific test conditions. No criterion on chicken consumption or salt consumption was enforced. Participants were informed beforehand that the activity consisted of tasting and eating chicken. Participants were briefed on the procedure, and written informed consent was obtained prior to participation. Ethical approval for the study was obtained from the Faculty of Natural and Agricultural Science of the University of Pretoria (NAS017/2023). At the end of the session, the participants received a store voucher of R50.

Each participant tasted nine different samples (Table 1). *RB* treatment was assessed in both groups to compare the ratings of the standard salty chicken between the two groups. Each sample weighing 6.4–8.4 g (representing two chicken cubes) was served at a temperature of 58°C–66°C. The nine samples were presented to participants in a balanced order using a Williams Latin square design. Participants rated the samples for the intensity of sweetness, saltiness, sourness, bitterness, and overall flavor intensity on linear continuous scales with word anchors (*not intense* and *very intense*) at the extreme ends. After this task was completed for the nine samples, a new set of nine samples was served to participants in a balanced order using a Williams Latin square design. For this second service round, participants rated the gap between their appreciation of each sample and their preferred saltiness level on a linear continuous scale with the word anchors *Really not salty enough* and *Way too salty* at the two extreme ends and a middle anchor labeled *The salt level is just right* (JAR task). The questionnaire and data collection were monitored using Compusense software (Version 23.0.27 2023/06/20).

### 2.5. Data Analysis

The intensity rating data were analyzed by two-way analysis of variance (ANOVA) on taste or flavor intensity using linear mixed-effect (LME) models (ranked-transformed data). Panelists were considered random factors, and the seasoning procedure (combining the salting level, the moment of salting, and the potential addition of flavorings) and the session number were considered fixed factors. Multiple comparisons between medians were performed using Wilcoxon tests when one factor was significant (*p* < 0.05). Wilcoxon tests were performed with a Bonferroni correction for multiple comparisons.

For the first group (treatments *Unsalted*, *RB*, *RBR*, *RBB*, *RBG*, *RP*, *RPR*, *RPB*, and *RPG*; Figure 1), the results from 12 participants were removed because all the samples were rated at 0 for saltiness intensity. For the second panel (treatments *RB*, *LB*, *LBR*, *LBB*, *LBG*, *LP*, *LPR*, *LPB*, and *LPG*; Figure 1), the results from five participants were removed for the same reason.

The OITE [25] was calculated for each flavoring treatment (treatments *RBR*, *RBB*, *RBG*, *RPR*, *RPB*, *RPG, LBR*, *LBB*, *LBG*, *LPR*, *LPB*, and *LPG*; Figure 1). OITE corresponded, for each panelist, to the difference between (i) the saltiness of the treatment containing an aroma and (ii) the saltiness of the unflavored solution that contained the same amount of sodium chloride and was salted following the same method (in broth or on the plate). We specifically tested whether, for each *salt level* × *salting time* × *flavoring* condition, the OITE means were different from 0 (one sample *t*-test).

For the JAR task, the raw data ranged from 0 to 10. For more straightforward plots and interpretations, all ratings were subtracted by 5. Then, median comparison tests were performed.

All the statistical analyses were performed using RStudio Software Version 4.1.2 [39]. LME models were generated using the function lme() in the nlme R package (v3.1-153; [40]), ANOVA and Wilcoxon tests were performed using the functions Anova() and wilcox_test() in the rstatix R package (v0.7.0; [41]), Welch two-sample *t*-tests were performed using the function t.test() in the stats R package [39], and median comparison tests were performed using the function Median.test() in the agricolae R package (v1.3-5; [42]). The effects were considered significant when *p* < 0.05.

## 3. Results

### 3.1. Sodium Content

Table 2 shows the sodium content of unsalted boiled chicken (*Unsalted*) boiled chicken salted at the regular level in the broth (*RB*) and boiled chicken salted at the low level in the broth (*LB*). There is a 33% difference in terms of sodium content between the two levels of salting (*RB* and *LB* treatments; Table 2).

**Figure 2:**
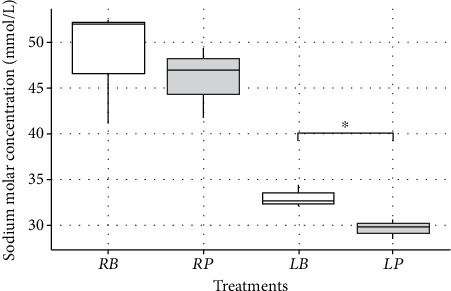
Sodium molar concentrations (millimoles per liter) measured by IC of supernatant collected after mixing and centrifugation of boiled chicken, depending on the level of salting and the moment of salting. *RB*, regular broth; *RP*, regular plate; *LB*, low broth; *LP*, low plate. Stars indicate the level of statistical significance for mean differences (Welch two sample *t*-test): ⁣^∗^*p* < 0.05.

The moment of salt addition (in broth or on the plate) marginally influenced the sodium concentration in the supernatant analyzed by IC at the regular level of salting (*p* = 0.07) (Figure 2). The sodium concentration at the low level was significantly lower when salt was added to the plate (*p* = 0.02), representing a reduction of approximately 10% (Figure 2).

Flavoring did not significantly affect the sodium concentration in the supernatant analyzed by IC (*p* = 0.73; Table 3).

### 3.2. Sensory Evaluations

The unsalted treatment was described as very low in sweetness, saltiness, sourness, bitterness, and overall flavor intensity (see Figure S1). The effects of flavorings on perceived sweetness, sourness, bitterness, and overall aroma are presented in Figures S2–S5.

#### 3.2.1. Absence of the Participant Group Effect

Boiled chicken salted at the regular level in the broth (*RB*) was evaluated by the two groups of participants. The mean saltiness intensity was not different between the two groups (*p* = 0.73; Table 4). The means of the JAR ratings were not significantly different (*p* = 0.08; Table 4). Wilcoxon test revealed that the medians of the JAR ratings were not significantly different either (*p* = 0.17; Table 4). Therefore, we conclude that there were no differences in the perception of saltiness between the two groups. Thus, one single global group will be considered for the rest of the study.

#### 3.2.2. Saltiness Intensity Ratings

##### 3.2.2.1. Effect of Salting Time on Saltiness Intensity

**Figure 3:**
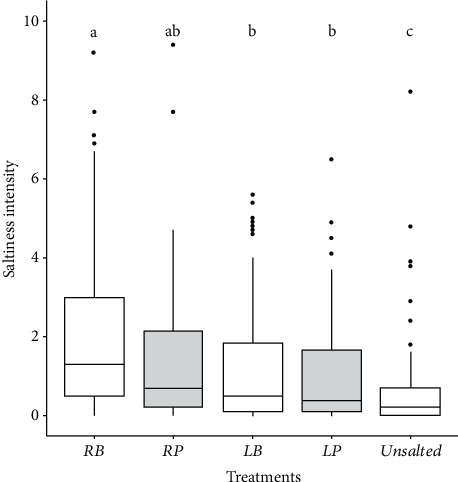
Salt intensity of chicken samples salted at either a regular salt level (*RB* and *RP* treatments) or a low salt level (*LB* and *LP* treatments) and salted either in broth (*RB* and *LB* treatments) or at the plate (grey boxes, *RP* and *LP* treatments). Treatments with the same letter are not significantly different (Wilcoxon test with Bonferroni correction for multiple comparisons, *p* < 0.05). *RB*, regular broth; *RP*, regular plate; *LB*, low broth; *LP*, low plate.

Figure 3 shows the saltiness intensity of unflavored treatments treated with salt in broth (*RB* and *LB* treatments), or on the plate (striped boxes, *RP* and *LP* treatments), at the regular salt level (*RB* and *RP* treatments) or at the low level (*LB* and *LP* treatments). Salting in broth was not significantly different from salting on the plate for the two levels of salting (there were no significant differences between *RB* and *RP* or between *LB* and *LP*; *p* > 0.2; Figure 3). The saltiness intensity of the chicken salted at the regular level at the plate (*RP*) was not significantly different from the saltiness intensity of the chicken salted at the low level in broth and at the plate (*LB* and *LP*) (Figure 3). The saltiness intensities of the salt treatments were all significantly different from the saltiness intensity of the *Unsalted* treatment (Figure 3).

##### 3.2.2.2. Effect of Flavorings on Saltiness Intensity

**Figure 4:**
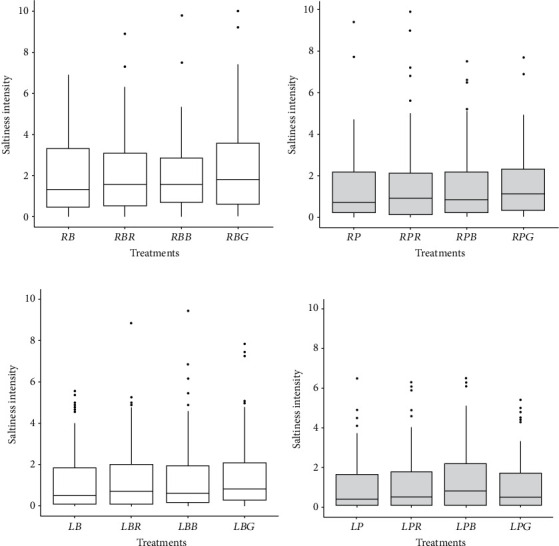
Mean saltiness intensity of chicken samples salted at the regular level in (a) broth and on the (b) plate or salted at the low level in (c) broth and on the (d) plate, either unflavored *RB*, *RP*, *LB*, and *LP* treatments or flavored with rosemary (*RBR*, *RPR*, *LB*R, and *LPR* treatments), smoked bacon (*RBB*, *RPB*, *LBB*, and *LPB* treatments), or smoked garlic (*RBG*, *RPG*, *LBG*, and *LPG* treatments). Results from ANOVA did not show any significant effect (*p* > 0.05) between samples.

At the two levels of salting (regular and low), the saltiness intensity was not significantly different (*p* > 0.05) between the unflavored and the flavored samples (Figure 4) regardless of when salt was added (in broth or on the plate).

**Figure 5:**
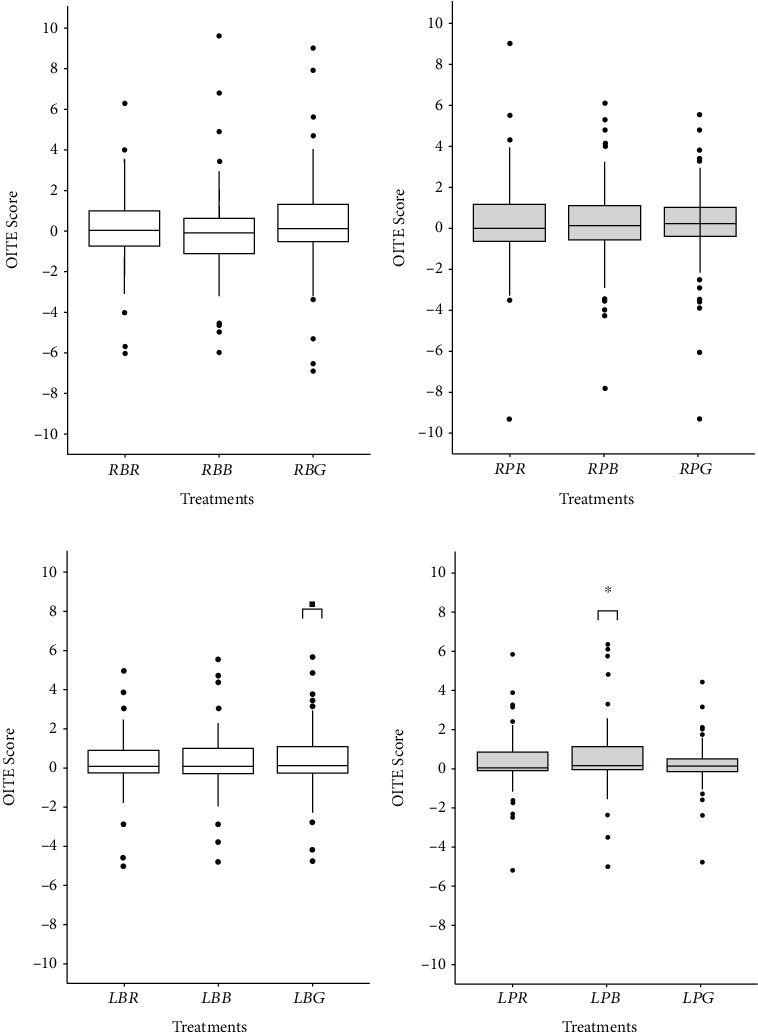
Odor-induced taste enhancement (OITE) scores for samples salted at the regular level in (a) broth and at the (b) plate or salted at the low level in (c) broth and at the (d) plate and flavored with rosemary (*RBR*, *RPR*, *LBR*, and *LPR* treatments), smoked bacon (*RBB*, *RPB*, *LBB*, and *LPB* treatments), or smoked garlic (*RBG*, *RPG*, *LBG*, and *LPG* treatments). OITE was calculated as the average across participant's scores, obtained by subtracting the score for the unflavored treatment from the score obtained for the respective flavored treatments. Stars indicate the level of statistical significance for mean differences compared to 0 (one sample *t*-test): ., *p* < 0.1; ∗, *p* < 0.05.

The OITE scores for the flavored treatments were not significantly different from 0 at the regular salt level (Figure 5a,b). However, at the low salt level, OITE was significantly different from 0 for smoked bacon when salting was performed on the plate (treatment *LPB*; *p* = 0.02; Figure 5d), and OITE was marginally significant for smoked garlic when salting was performed in broth (treatment *LBG*; *p* = 0.07; Figure 5c).

#### 3.2.3. JAR Saltiness

**Figure 6:**
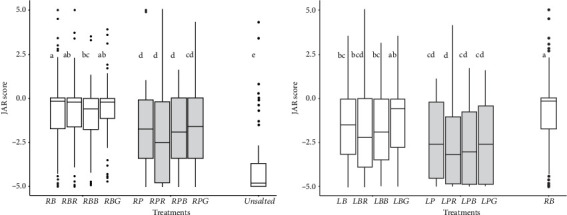
JAR scores (a) for chicken samples salted at the regular level and the *Unsalted* treatment and (b) for samples salted at the low level and the control sample *RB* salted at the regular level in broth. Chicken samples were flavored with rosemary (*RBR*, *RPR*, *LBR*, and *LPR* treatments), smoked bacon (*RBB*, *RPB*, *LBB*, and *LPB* treatments), or smoked garlic (*RBG*, *RPG*, *LBG*, and *LPG* treatments), or unflavored (*RB*, *RP*, *LB*, and *LP* treatments) and were salted either in broth (white boxes) or at the plate (grey boxes). Treatments with the same letter are not significantly different at *p* < 0.05 (median comparison test).

In the JAR task, a score of −5 indicates that the sample is clearly not salty enough, a score of 0 indicates that the sample is just right in terms of salty taste, and a score of +5 indicates that the sample is overly salty. To check for significant differences between conditions, the following median comparison tests were performed: one included the treatments *Unsalted*, *RB*, *RBR*, *RBB*, *RBG*, *RP*, *RPR*, *RPB*, and *RPG* (Figures 1 and 6a), and the other included the *RB*, *LB*, *LBR*, *LBB*, *LBG*, *LP*, *LPR*, *LPB*, and *LPG* treatments (Figures 1 and 6b). For each test, a common letter indicates that the treatments are not significantly different.

The results presented in Figure 6a showed that boiled chicken with no salt added (*Unsalted*) was, as expected, not salty enough. The median of the chicken samples salted in the broth at the regular level (*RB*) was approximately 0, indicating a JAR salty taste regardless of the flavoring conditions. The median number of chicken samples salted at the plate at the regular level (*RP*) was less than 0 regardless of the flavoring conditions, which indicates that these chicken pieces were not salty enough. The median comparison revealed that the JAR scores were significantly different when salt was added to the broth and on the plate except for *RBB* (chicken flavored with smoked bacon in the broth), for which the JAR score was not significantly different than that of samples salted on the plate and flavored with smoked garlic.

The results depicted in Figure 6b indicate that except for the sample salted at the low level in broth with smoked garlic (*LBG*), the chicken samples salted at the low salt level were perceived as less salty than the control sample at the regular salt level (*RB*). Interestingly, this sample attained a mean JAR very close to 0 despite containing approximately 30% less salt, suggesting that the sample was perceived as adequately salted. In contrast, all other chicken samples received a JAR score below 0, indicating that they were considered under salted. Furthermore, the samples salted at the plate were consistently judged as less salty than those salted in broth, with only the rosemary-flavored sample showing a significantly lower JAR score—a trend consistent with observations at the regular salt level.

## 4. Discussion

In this study, we investigated the influence of various flavoring and salting practices on the perceived saltiness of boiled chickens. We explored the following methods of adding discretionary salt: in broth during boiling and on the plate after cooking. Our hypothesis suggested that adding salt to the plate causes salt crystals to remain on the meat's surface, leading to a more heterogeneous salt distribution and enhancing salty taste intensity. Additionally, we applied three flavor treatments—rosemary, smoked bacon, and smoked garlic—to the broth, aiming to enhance the perception of saltiness through odor stimulation.

The results revealed no significant differences in perceived saltiness intensity between the two salting practices. Compared to adding salt to the broth as the chicken boils, adding discretionary salt to the plate after cooking did not lead to enhanced salt perception. This result was obtained across both salt levels investigated. Our hypothesis was that adding salt to the plate leads to a heterogeneous distribution of salt due to the persistence of salt crystals or salt-concentrated spots on the food surface. In contrast, adding salt to the broth during boiling may facilitate the penetration of salt into the meat, resulting in a more homogeneous salt distribution. However, high temperatures (> 65°C) during cooking increase the firmness of muscular tissues and cause tissue contraction [43]. This contraction likely limits the diffusion of sodium ions from the broth into the meat, causing them to remain primarily at the periphery of the food. This mechanism could explain why the difference in salt distribution between the two salting methods might be less significant than initially anticipated. A similar study on vegetables or starchy foods, such as pasta, where sodium ions can more readily penetrate the food matrix during cooking, might yield valuable insights. For example, Bianchi et al. [44] observed a linear relationship between the salt concentration in water and the sodium content in cooked pasta, suggesting that sodium ions can effectively penetrate this type of food. These findings underscore the importance of understanding the interaction between food matrix composition, cooking method, and sodium distribution in shaping salt perception.

Regarding the impact of aroma addition, our results showed that rosemary, smoked garlic, and smoked bacon flavorings did not significantly influence the perceived intensity of salt at the regular salt level. However, at the 33% reduced salt level, a limited yet significant odor-induced saltiness enhancement was observed when smoked bacon flavoring was added to the broth and salt was added to the plate. Moreover, a marginally significant increase in saltiness was observed with smoked garlic when salt was added to the broth. This trend was further supported by the JAR results, which indicated that the addition of smoked garlic flavoring to the broth, in which salt was added, was perceived as almost just right in terms of saltiness and noticeably similar to the unflavored cooked chicken salted at the regular level in broth. This finding confirms that sodium levels can be reduced by 30% without compromising the flavor by adding aroma compounds, such as smoked garlic, to the broth when cooking chicken. Therefore, our results confirm that OITE is an effective mechanism to compensate for salt reduction in food [24, 25, 37, 38]. It would be interesting to investigate the specific odor-active compounds in smoked garlic responsible for the strong odor-induced saltiness enhancement. Future studies could explore whether this effect is driven primarily by the smoky compounds, the garlic-related compounds, or a combination of these compounds and those present in the broth. Moreover, this sensory mechanism has been demonstrated to be applicable in domestic cooking practices, with smoked garlic and smoked bacon flavorings showing promise in this regard. In contrast to a previous report [35], the rosemary flavor did not enhance saltiness under our conditions, possibly because chickens flavored with rosemary were perceived to be more bitter and slightly sour than unflavored chickens or chickens flavored with other aromas (see Figures S3 and S4), which could have counteracted the increase in saltiness. Nevertheless, Mediterranean aromatic plants, including rosemary, have been used to flavor commercial sea salts, enabling a 15% reduction in salt content without decreasing saltiness intensity [33, 34]. Depending on the food items and recipe, using commercial flavored sea salts or direct flavorings could therefore offer an effective strategy for reducing salt intake. Additionally, it appears that odor-induced saltiness enhancement varies among different aromatic herbs [45], warranting further research to gain more insight into these differences.

A more significant OITE at lower salt contents was expected, given that this mechanism has been demonstrated to be more effective at low salt intensities (e.g., [25, 26]). However, the salt content of the samples used in the present study and the corresponding salty taste intensity ratings were both quite low, even at the regular level, despite being adequate within the JAR evaluation. Moreover, the lack of significant results at regular and sometimes low salt levels might be attributed to the low odor intensity of the added flavorings, which may not be clearly perceived by participants (see Figure S5). Although previous researchers did not find a clear correlation between the intensity of odor and the amplitude of OITE [46], this possibility should be further addressed through dedicated studies.

In this study, we combined known strategies to compensate for taste issues in food with reduced salt content and applied the methods to domestic cooking and seasoning practices. Emorine et al. [12] successfully achieved heterogeneous salt distribution and odor-induced saltiness enhancement in a hot-served snack. They designed a ham-flavored snack with 35% less salt, which was perceived as saltier and was liked equally as standard snacks. Similarly, in the present study, salt was added to plates to achieve heterogeneous salt distribution, and smoked bacon flavoring was added to effectively enhance saltiness at low salt contents. Interestingly, statistical significance in terms of enhanced salty tastes was only achieved by the combination of these two strategies. Although further research is needed to clarify why the combined effect was more pronounced, combined strategies should be employed to more efficiently address taste issues in low salt foods and to improve cooking and seasoning practices, thereby contributing to a reduction in salt intake.

Regarding the limitations of the present study, each participant tasted 18 samples within a 1 h session. This process could have been overwhelming for some participants and might have led to inconsistent sensory ratings, thereby increasing the variability of the collected data. Additionally, for practical reasons, the amount of salt added to the plate was estimated based on the cooked chicken mass and weighed before cooking began. This constraint should be considered when comparing the two salting conditions (in broth or on the plate). One outcome of this procedure was that the amount of salt added to the plate was approximately 10% lower than the quantity added to the broth. This difference may have contributed to the sensory results showing a systematically lower salty taste intensity for the samples salted at the plate and could have reduced the magnitude of the observed effect. The salt added to the plate could have been more precisely measured if the cooked mass of each batch of chicken had been determined. In this study, we did not investigate the habits of participants regarding salt usage, such as whether they regularly added table salt or were interested in reducing salt intake or their preferences for salty foods or the food they prepared. Antúnez et al. [47] reported that consumers that added salt to foods less frequently gave higher overall liking scores to rice with 25%–50% salt reduction, while De Kock et al. [10] did not find any differences in liking ratings between consumers interested in lowering salt intake and those who were not. We did not assess if the participants liked the products. Instead, we used a JAR scale, which likely partly captured the hedonic dimension. Measuring the likeability would provide complementary data of interest, as it is a key determinant of food acceptability.

Furthermore, our findings are specific to boiled chicken. For other types of meat, boiling is often applied to cuts rich in connective tissues and typically involves longer cooking times. Extended cooking may allow the salt to penetrate more deeply into the food matrix, potentially resulting in significantly lower sodium availability for taste compared to salting on the plate. This could result in a more pronounced contrast in perceived saltiness between the two salting practices and might make salting on the plate more efficient for such cuts of meat. Regarding flavorings, it is plausible that smoky flavors could also enhance saltiness perception in other types of meat or even vegetables, particularly when these foods are roasted, as roasting often amplifies both flavor and aroma profiles.

## 5. Conclusion

In the context of culinary practices, our findings emphasize that the timing of salt addition—whether during cooking (e.g., in broth) or after cooking (e.g., on the plate)—has little impact on the perceived saltiness of chicken breast meat, specifically in situations where no flavorings were used. However, when combined with salt-enhancing flavorings, such as smoked garlic or smoked bacon, these practices offer a promising approach to reducing discretionary salt intake while preserving taste, particularly at lower salt levels. This finding is especially relevant in regions where meat is widely consumed, offering a strategy to address high salt intake by promoting the use of such flavorings in culinary practices. Furthermore, our study underscores the need to broaden research efforts to include diverse food items with varying compositions, structures, and textures. This comprehensive approach could provide deeper insights into salt perception and reduction strategies across a wide range of food products. Future research should focus on identifying the specific compounds responsible for saltiness enhancement in smoked flavorings, as well as exploring the effects of salt-enhancing flavorings in foods with different moisture levels, fat content, or complexity. These efforts hold significant potential to advance global initiatives aimed at reducing salt intake.

## Figures and Tables

**Table 1:** Tasting group characteristics.

**Tasting group**	**1**	**2**
Number of participants	82	76
Percentage of women	72%	70%
Mean age and calculated standard deviation	24 ± 4 years old	25 ± 5 years old
Tasted treatments	*Unsalted*, *RB*, *RBR*, *RBB*, *RBG*, *RP*, *RPR*, *RPB*, and *RPG*	*RB*, *LB*, *LBR*, *LBB*, *LBG*, *LP*, *LPR*, *LPB*, and *LPG*

**Table 2:** Sodium content of the *Unsalted*, *RB*, and *LB* treatments measured by ICP–MS.

**Treatment**	**Sodium content (mmol of Na** ^**+**^ **/100** g **of chicken)**
*Unsalted*	2.0
*RB*	6.5
*LB*	4.1

**Table 3:** Sodium concentration in the supernatant analyzed by IC for unsalted chicken either unflavored, flavored with smoked bacon, flavored with smoked garlic, or flavored with rosemary.

**Treatments**	**Sodium concentration (mmol/L)**
*Unsalted*	13.5 ± 1.8
*Unsalted* flavored with smoked bacon	14.0 ± 1.2
*Unsalted* flavored with smoked garlic	12.7 ± 0.7
*Unsalted* flavored with rosemary	12.5 ± 3.0

**Table 4:** Saltiness intensity scores and JAR ratings of the boiled chicken salted at the regular level in the broth (*RB* treatment) depending on the tasting group.

**Tasting group**	**Saltiness intensity (** **m** **e** **a** **n** ± **standard** **deviation)**	**JAR rating (mean)**	**JAR rating (median)**
1	2.06 ± 2.00	−0.4	0
2	1.94 ± 2.06	−1.0	−0.2

## Data Availability

The research data used to support the findings of this study are available from the corresponding author upon request.

## Supporting Information

Supporting Information Additional supporting information can be found online in the Supporting Information section. The supplementary file attached shows the sensory characteristics of the “Unsalted” treatment as well as the saltiness, sweetness, sourness, bitterness, and overall flavor intensities of all the tested samples.
