# Supplementary material for: Exploring Culinary Methods to Reduce Sodium Intake: The Impact of Flavorings and Salt Addition Timing in Boiled Chicken
Source: Int J Food Sci. 2025 Jun 6;2025:3703692. doi: 10.1155/ijfo/3703692 (PMC12165749; doi:10.1155/ijfo/3703692)
Supplement: Supporting Information — Additional supporting information can be found online in the Supporting Information section. The supplementary file attached shows the sensory characteristics of the “Unsalted” treatment as well as the saltiness, sweetness, sourness, bitterness, and overall flavor intensities of all the tested samples. [file 3703692.f1.docx]

# Appendix A. Supplementary data

## Characteristics of the ‘Unsalted’ treatment


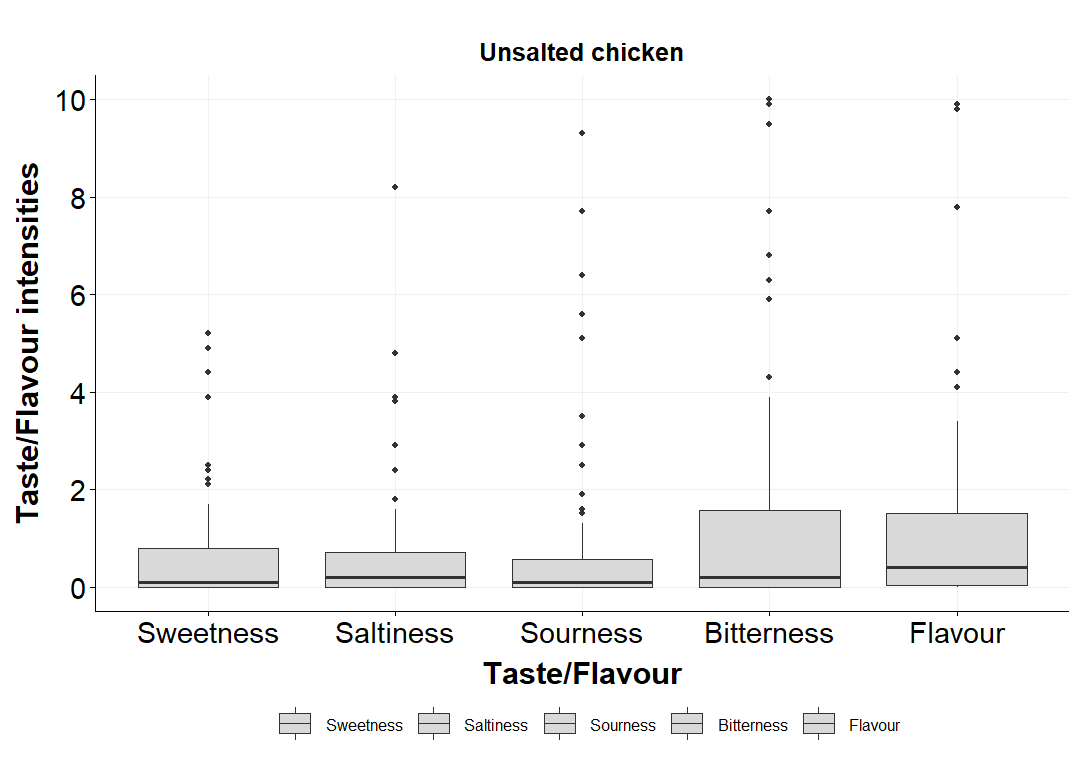


Figure S1: Sensory characteristics of the unsalted chicken sample

## Sweetness intensity


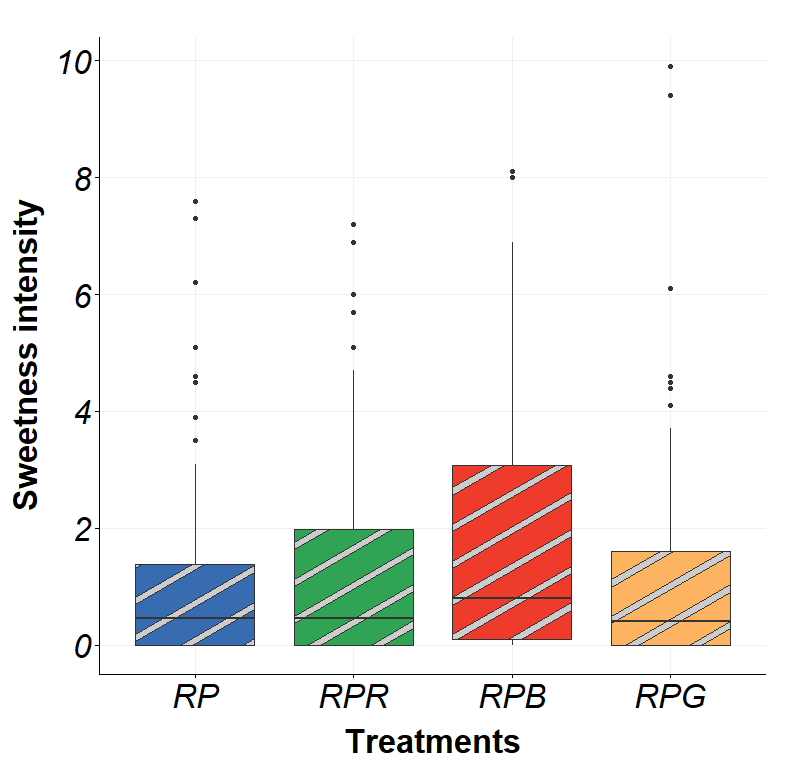

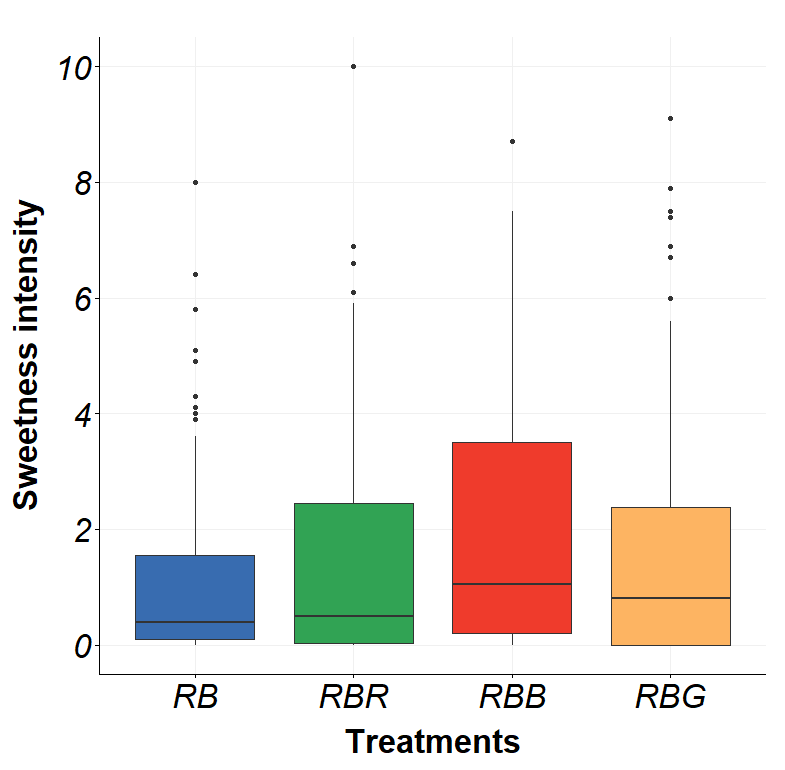

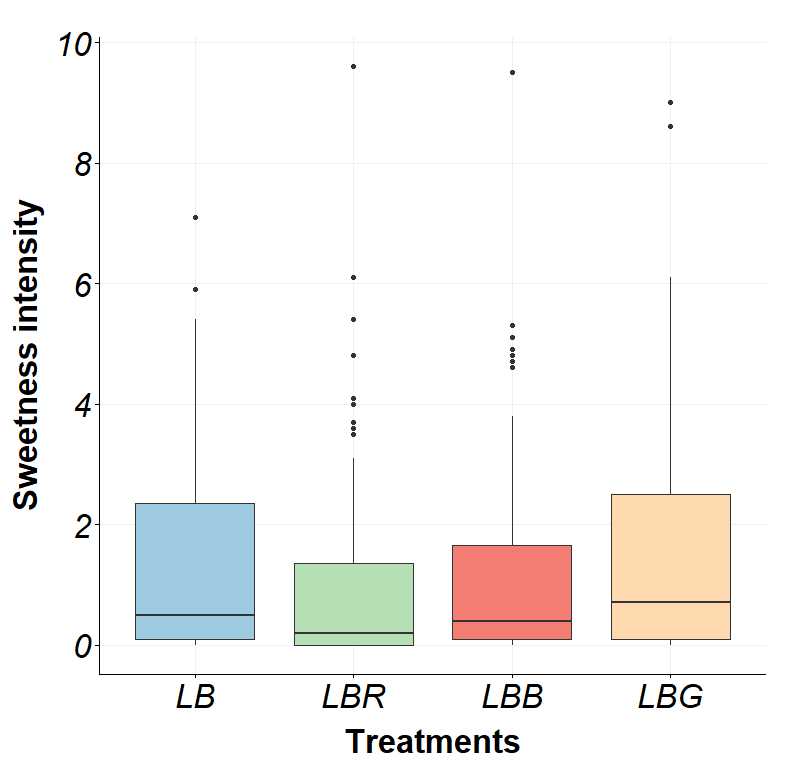

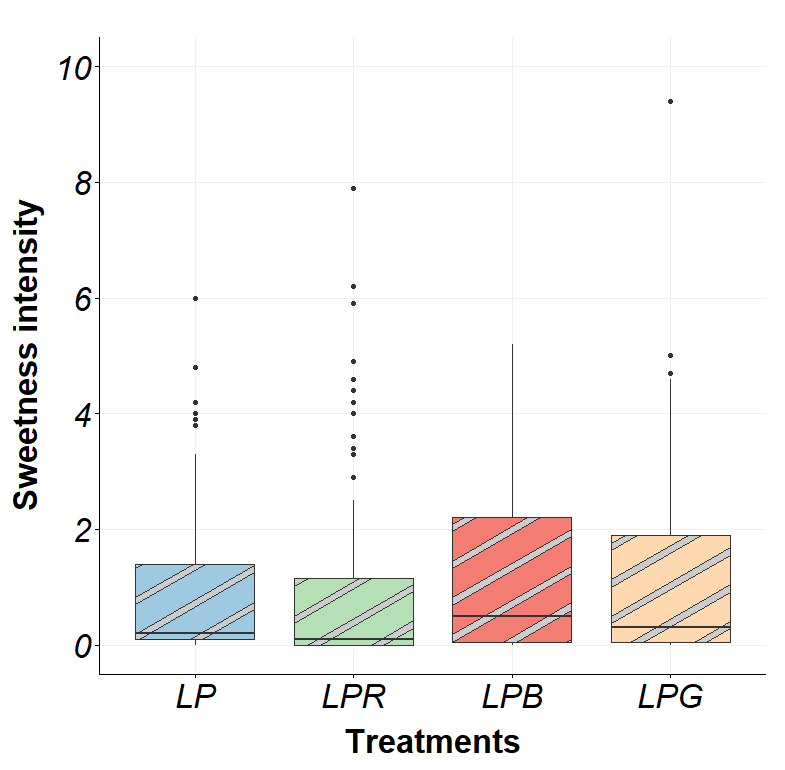


**A**

**C**

**B**

**D**

*Figure S2: Mean saltiness intensity of chicken samples salted at the Regular level in Broth (fig. S2.A) and at the Plate (fig. S2.B) or salted at the Low level in Broth (fig. S2.C) and at the Plate (fig. S2.D), either unflavoured (blue boxes) or flavoured with Rosemary (green boxes), smoked Bacon (red boxes) or smoked Garlic (orange boxes). Results from ANOVA on treatments RB, RBR, RBB and RBG showed a treatment effect (p-value = 0.02). Post-hoc analysis showed no significant differences but smoked bacon flavour tended to increase sweetness when salt was added in broth. Results from ANOVA on treatments LB, LBR, LBB and LBG showed a treatment effect (p-value = 0.003). Post-hoc analysis showed no significant differences between treatments. Results from ANOVA on treatments RP, RPR, RPB, RPG, and on treatments LP, LPR, LPB and LPG did not show any significant effect (p > 0.05).*

## Sourness intensity

**C**

**A**


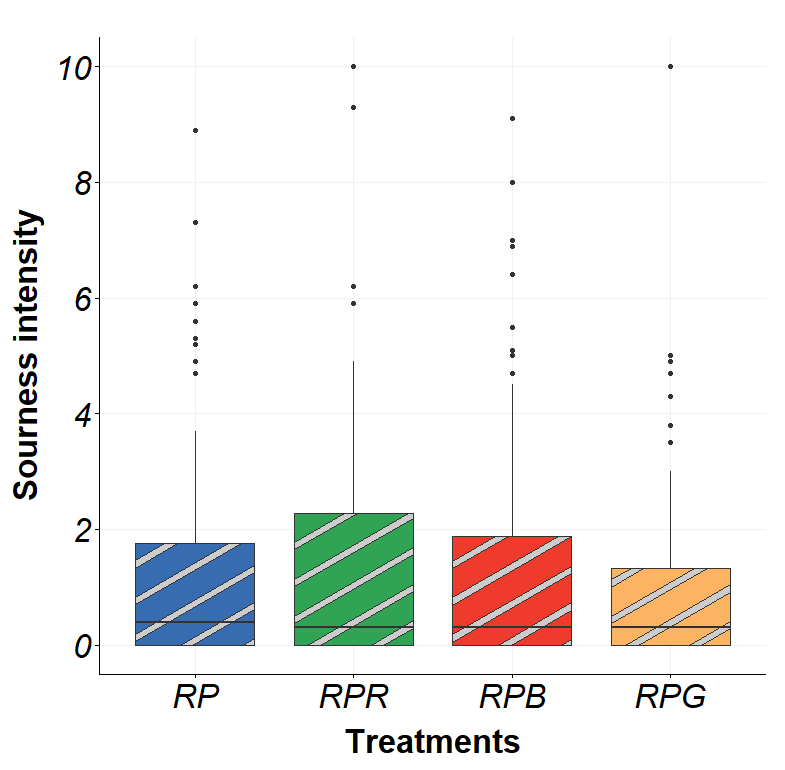

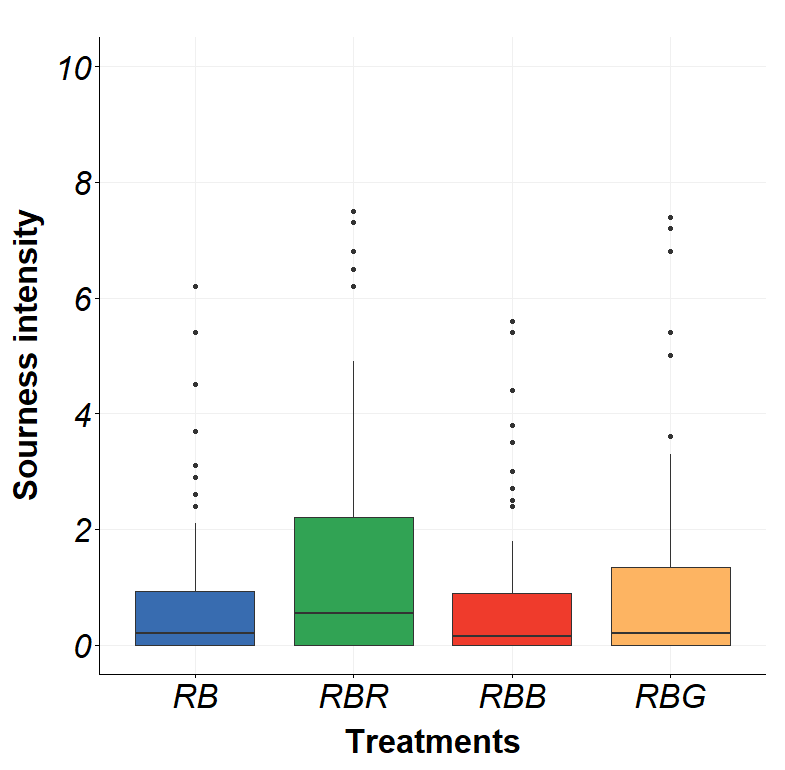


**D**

**B**


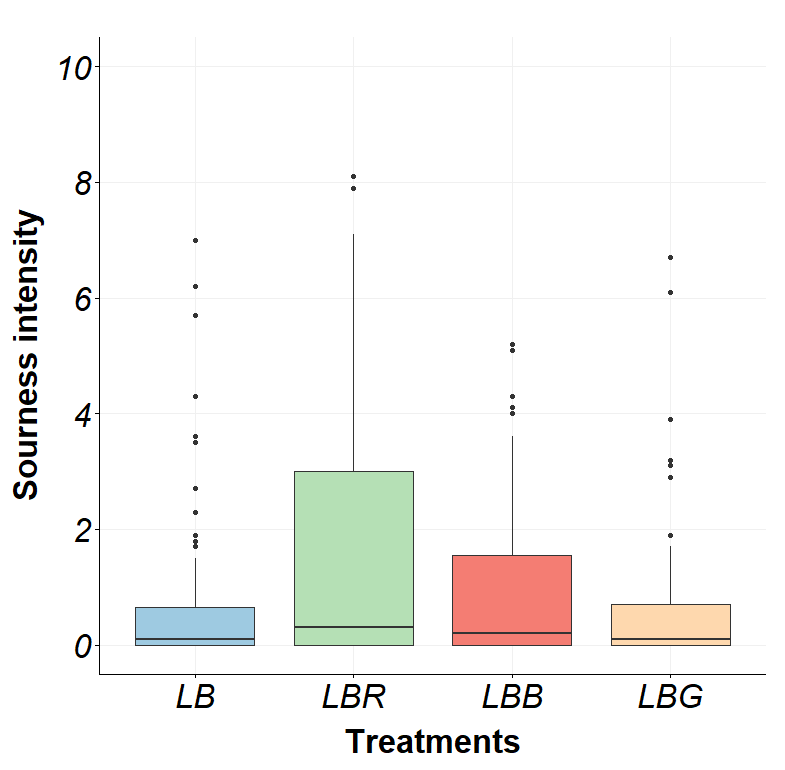

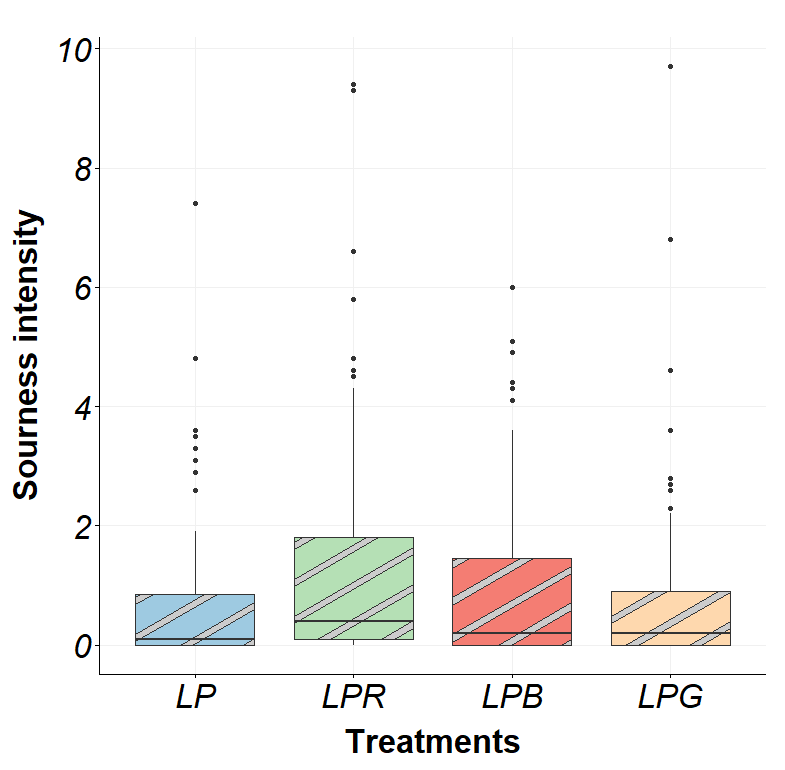


*Figure S3: Mean saltiness intensity of chicken samples salted at the Regular level in Broth (fig. S3.A) and at the Plate (fig. S3.B) or salted at the Low level in Broth (fig. S3.C) and at the Plate (fig. S3.D), either unflavoured (blue boxes) or flavoured with Rosemary (green boxes), smoked Bacon (red boxes) or smoked Garlic (orange boxes). The results from ANOVA on treatments RB, RBR, RBB and RBG showed a treatment effect (p-value = 0.01). Post-hoc analysis showed no significant differences between treatments. The result from ANOVA on treatments LB, LBR, LBB and LBG showed a treatment effect (p-value = 0.009). Post-hoc analysis showed no significant differences between treatments. The result from ANOVA on treatments LP, LPR, LPB and LPG showed a treatment effect (p-value < 0.001). Post-hoc analysis showed no significant differences between treatments. Results from ANOVA on treatments RP, RPR, RPB and RPG did not show any significant effect (p > 0.05).*

## Bitterness intensity

**C**

**A**


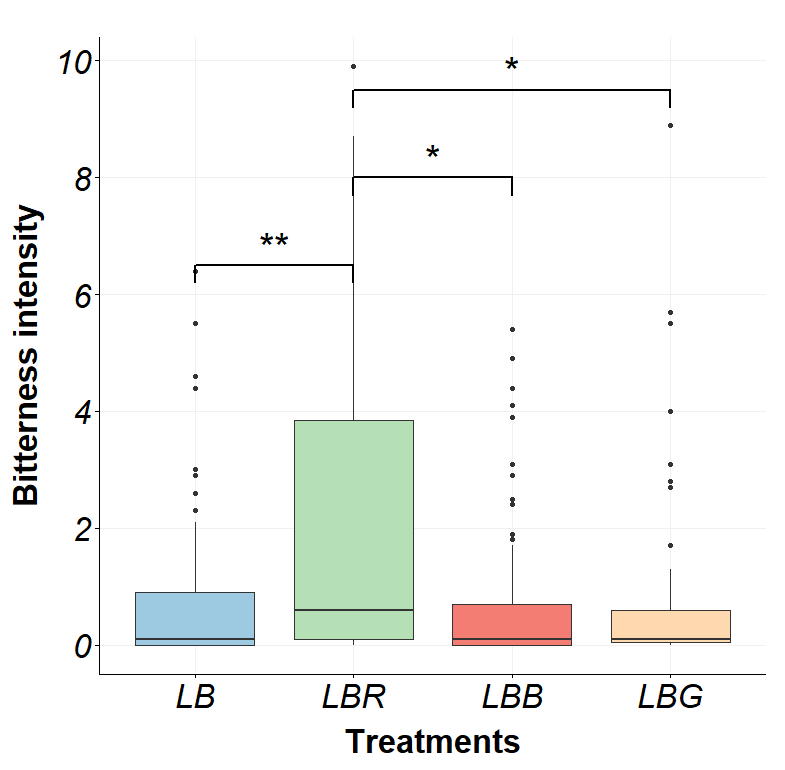

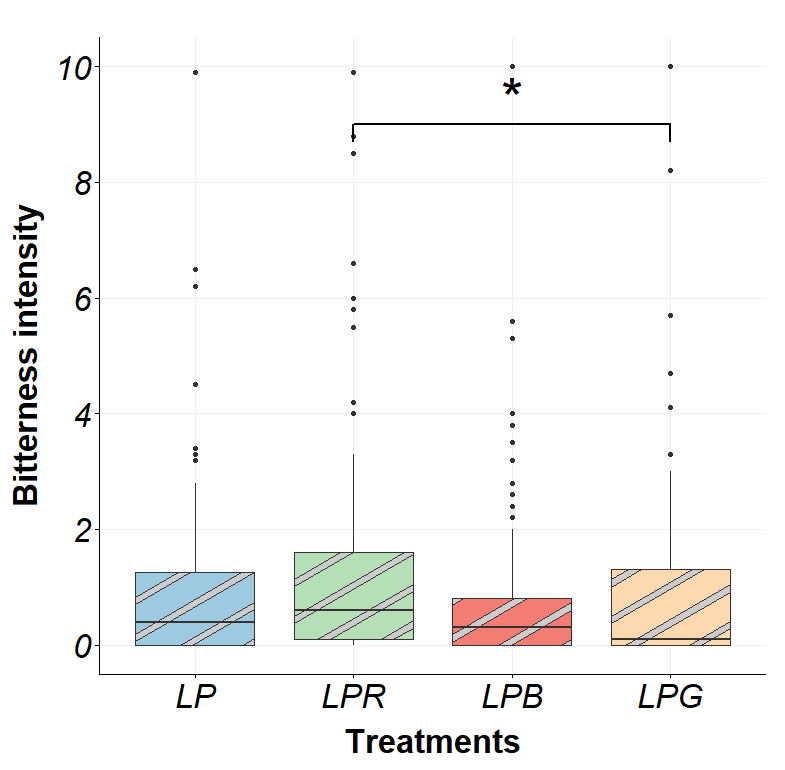


**D**

**B**


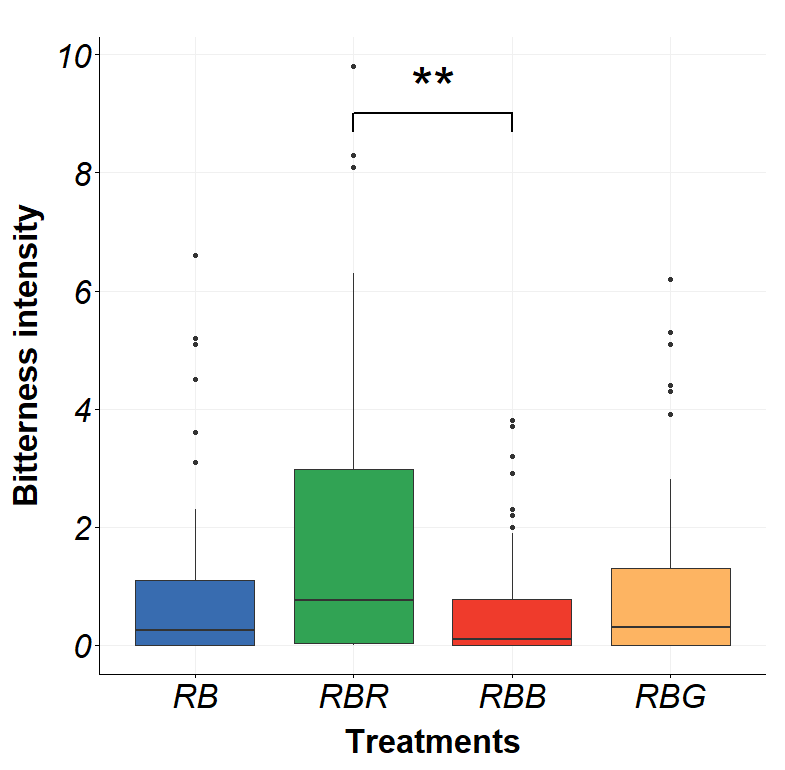

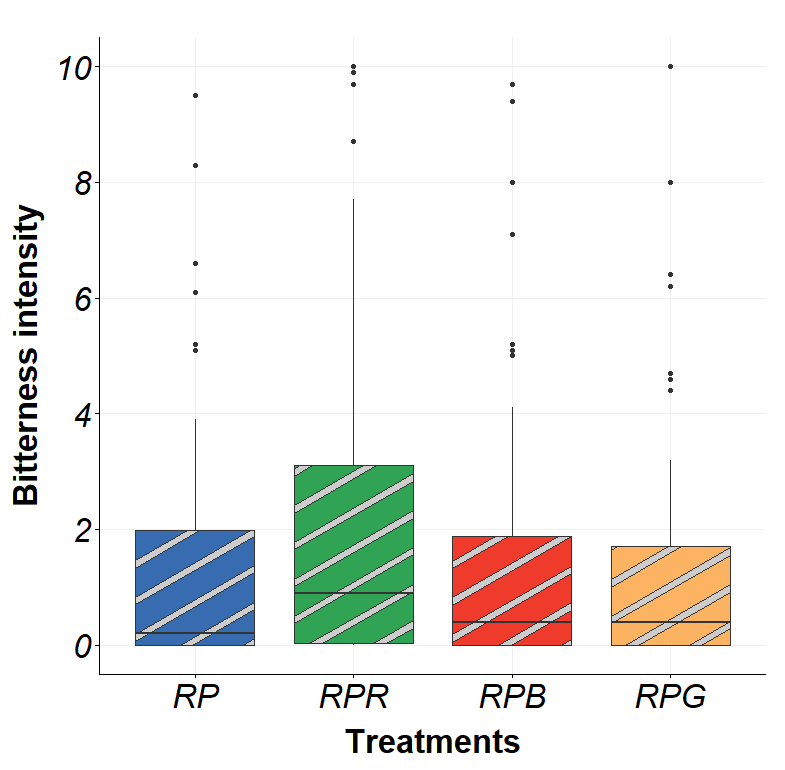


*Figure S4: Mean saltiness intensity of chicken samples salted at the Regular level in Broth (fig. S4.A) and at the Plate (fig. S4.B) or salted at the Low level in Broth (fig. S4.C) and at the Plate (fig. S4.D), either unflavoured (blue boxes) or flavoured with Rosemary (green boxes), smoked Bacon (red boxes) or smoked Garlic (orange boxes). The results from ANOVA on treatments RB, RBR, RBB and RBG showed a treatment effect (p-value < 0.001). Post-hoc analysis showed that chicken with rosemary aroma was perceived as more bitter than chicken flavoured with smoked bacon. The results from ANOVA on treatments RP, RPR, RPB and RPG showed a treatment effect (p-value = 0.01). Post-hoc analysis showed no significant differences between treatments. The results from ANOVA on treatments LB, LBR, LBB and LBG showed a treatment effect (p-value < 0.001). Chicken salted in broth and with rosemary was significantly more bitter than the other treatments. The results from ANOVA on treatments LP, LPR, LPB and LPG showed a treatment effect (p-value < 0.001). Chicken salted in broth and with rosemary was significantly more bitter than when smoked garlic was used.*

## Flavour intensity

**D**

**C**

**B**

**A**


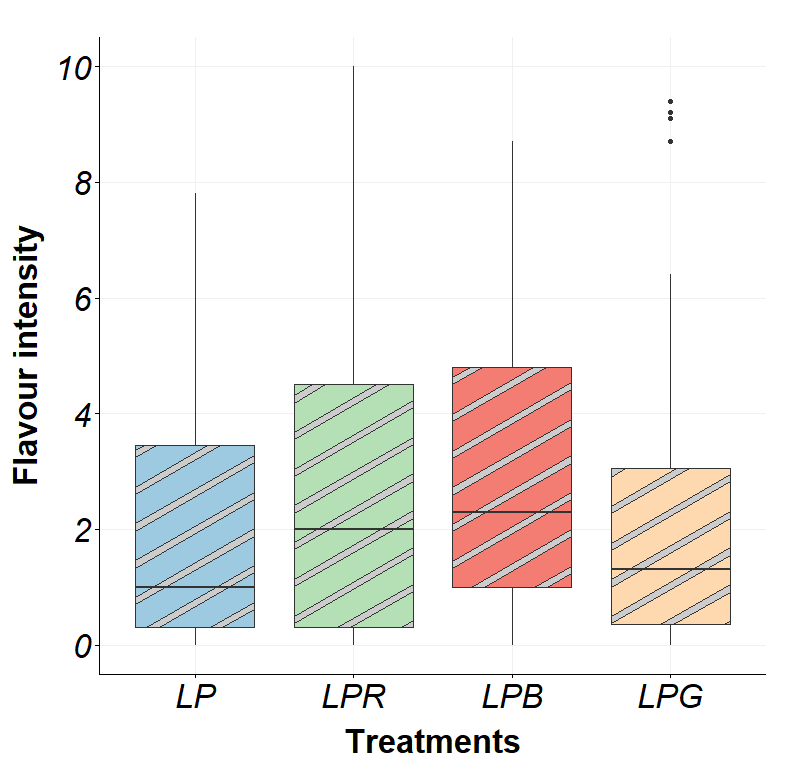

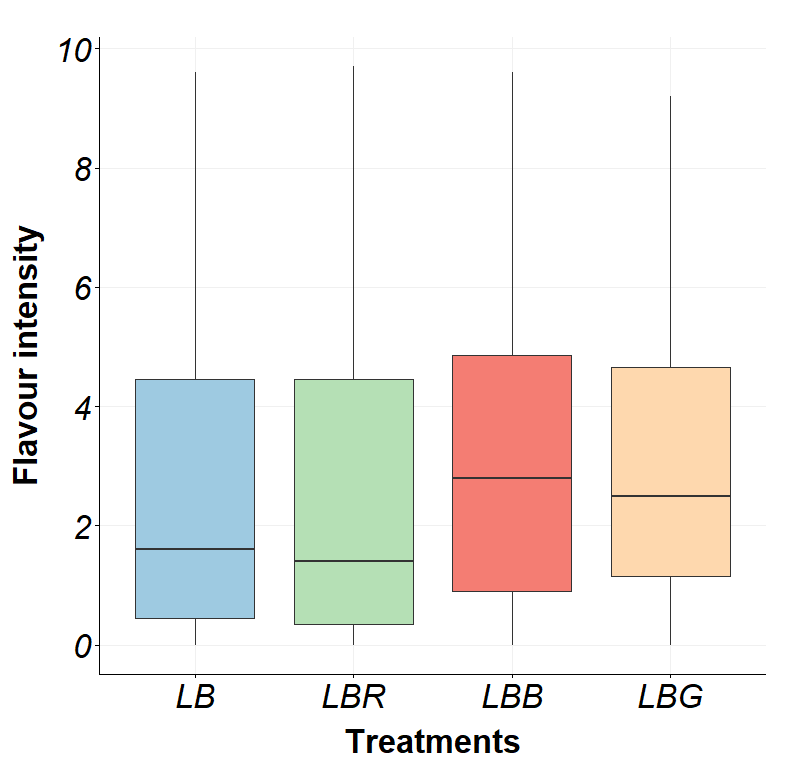

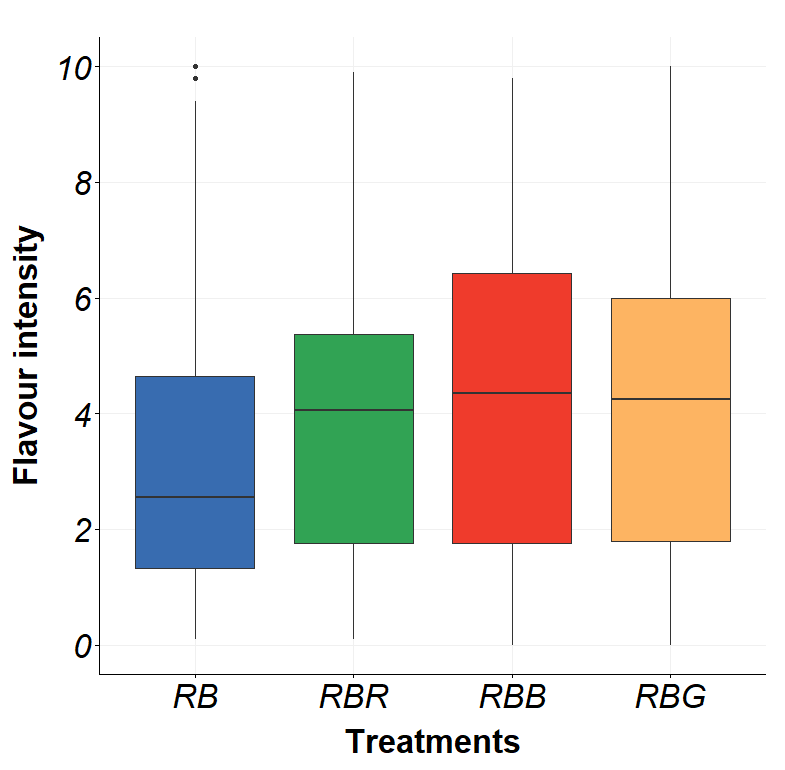

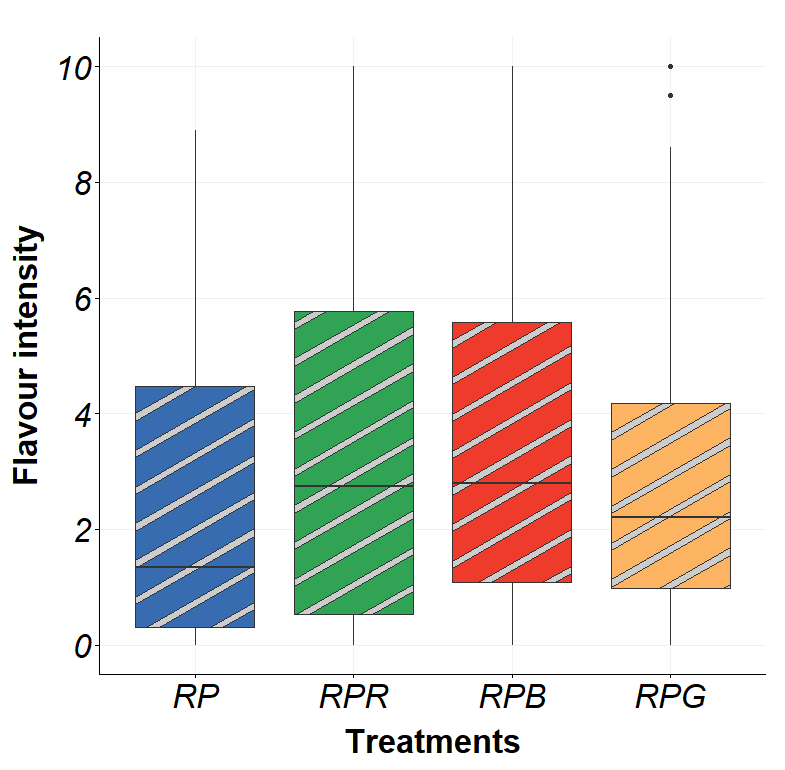


*Figure S5: Mean saltiness intensity of chicken samples salted at the Regular level in Broth (fig. S5.A) and at the Plate (fig. S5.B) or salted at the Low level in Broth (fig. S5.C) and at the Plate (fig. S5.D), either unflavoured (blue boxes) or flavoured with Rosemary (green boxes), smoked Bacon (red boxes) or smoked Garlic (orange boxes). The results from ANOVA on treatments RB, RBR, RBB and RBG showed a treatment effect (p-value = 0.01). Post-hoc analysis showed no significant differences between treatments. The results from ANOVA on treatments RP, RPR, RPB and RPG showed a treatment effect (p-value = 0.02). Post-hoc analysis showed no significant differences between treatments. The results from ANOVA on treatments LB, LBR, LBB and LBG showed a treatment effect (p-value = 0.003). Post-hoc analysis showed no significant differences between treatments. The results from ANOVA on treatments LP, LPR, LPB and LPG showed a treatment effect (p-value = 0.01). Post-hoc analysis showed no significant differences between treatments.*
